# The “Loopole” Antenna: A Hybrid Coil Combining Loop and Electric Dipole Properties for Ultra-High-Field MRI

**DOI:** 10.1155/2020/8886543

**Published:** 2020-09-07

**Authors:** Karthik Lakshmanan, Martijn Cloos, Ryan Brown, Riccardo Lattanzi, Daniel K. Sodickson, Graham C. Wiggins

**Affiliations:** 1 Bernard and Irene Schwartz Center for Biomedical Imaging, Department of Radiology, New York University Grossman School of Medicine, New York, NY, USA; 2 Center for Advanced Imaging Innovation and Research (CAI2R), Department of Radiology, New York University Grossman School of Medicine, New York, NY, USA; 3 Tech4Health, NYU Langone Health, New York, NY, USA

## Abstract

**Purpose.:**

To revisit the “loopole,” an unusual coil topology whose unbalanced current distribution captures both loop and electric dipole properties, which can be advantageous in ultra-high-field MRI.

**Methods.:**

Loopole coils were built by deliberately breaking the capacitor symmetry of traditional loop coils. The corresponding current distribution, transmit efficiency, and signal-to-noise ratio (SNR) were evaluated in simulation and experiments in comparison to those of loops and electric dipoles at 7 T (297 MHz).

**Results.:**

The loopole coil exhibited a hybrid current pattern, comprising features of both loops and electric dipole current patterns. Depending on the orientation relative to B_0_, the loopole demonstrated significant performance boost in either the transmit efficiency or SNR at the center of a dielectric sample when compared to a traditional loop. Modest improvements were observed when compared to an electric dipole.

**Conclusion.:**

The loopole can achieve high performance by supporting both divergence-free and curl-free current patterns, which are both significant contributors to the ultimate intrinsic performance at ultra-high field. While electric dipoles exhibit similar hybrid properties, loopoles maintain the engineering advantages of loops, such as geometric decoupling and reduced resonance frequency dependence on sample loading.

## 1. Introduction

Compared to conventional magnetic resonance imaging (MRI) systems, ultra-high-field (UHF) MRI systems (defined as those with B_0_ field strength of 7 T and above) promise an enhanced signal-to-noise ratio (SNR) [1–3]. However, UHF MRI also presents a variety of challenges, some of which are related to accentuated interactions between the applied radiofrequency (RF) magnetic field $\left({\text{B}}_{\text{1}}^{\text{+}}\right)$ and the dielectric biological tissues. For example, since at UHF, the RF wavelength becomes comparable to the dimensions of the human body, undesirable interference patterns emerge that lead to inhomogeneous excitations [4–6]. At the same time, stronger eddy currents in conductive tissues result in increased RF power deposition, which is a safety concern.

At the center of these phenomena is the RF coil, which plays the crucial role of delivering the excitation to the body and detecting the induced signal from the body. Much recent work has been performed to optimize RF coils in order to fully realize the potential of UHF MRI. Using the far field antenna theory, it was shown that a radiative dipole can be an effective UHF transmit/receive element under certain conditions related to the operating frequency and sample properties [7, 8]. The radiative dipole represented a drastic change from conventional closed loop coils and stripline elements [9–12], which had been ubiquitous in MRI. The experimental findings by Raaijmakers and colleagues [7, 8] were in agreement with concurrent theoretical work on ideal current patterns, which provided insight on how to sculpt actual coils that approached ultimate performance [13]. In particular, ideal current patterns analysis showed that “loop-like” currents alone could saturate optimal performance at the center of a body-size sample only at low field strength, while “electric-dipole-like” currents become substantial contributors at UHF [14–18].

Here, we define electric-dipole-like currents as those with a source and a sink, while loop-like currents refer to those with a closed return path (i.e., divergence-free current patterns). Note that electric dipole antennas are comprised of both divergence-free and curl-free current components [19], whereas the currents in typical loop antennas are purely divergence-free.

Insights obtained from far field antenna theory combined with the intriguing observation that closed and nonclosed current paths both contribute to the optimal performance at UHF inspired mixed arrays consisting of loop/dipole combinations, which provided significant SNR improvements compared to arrays of loops or dipoles alone [19–22].

The current work builds on the desire to conceive an array with a dual nature that captures both loop and dipole properties. Conventional RF loop coils have been designed to maintain approximately uniform current amplitude around their perimeter. While this has been put diligently into practice for decades by symmetrically distributing capacitors around loops, it coincidentally suppresses possible beneficial electric-dipole-like currents. Here, we revisit, instead, the possibility of deliberately creating a nonuniform current distribution to capture both loop-like and electric-dipole-like currents with a single resonant loop element. We refer to this hybrid element as the “loopole,” which was introduced in an abbreviated conference abstract in 2014 [23] and later demonstrated as a means for coil decoupling [24]. The aim of this work is to review, in more detail, the loopole fundamental properties and performance.

## 2. Methods

In conventional loop coil design, it is common practice to pursue both uniform current distribution and robustness against load sensitivity. This is achieved by placing multiple capacitors of equal value at equal intervals about the perimeter of the loop. The resulting loop is characterized by having a completely closed current path, which is analogous to a magnetic dipole, and has been shown to represent the ideal receive element to maximize SNR at any depth at low frequencies [13, 15].

**Figure 1:**
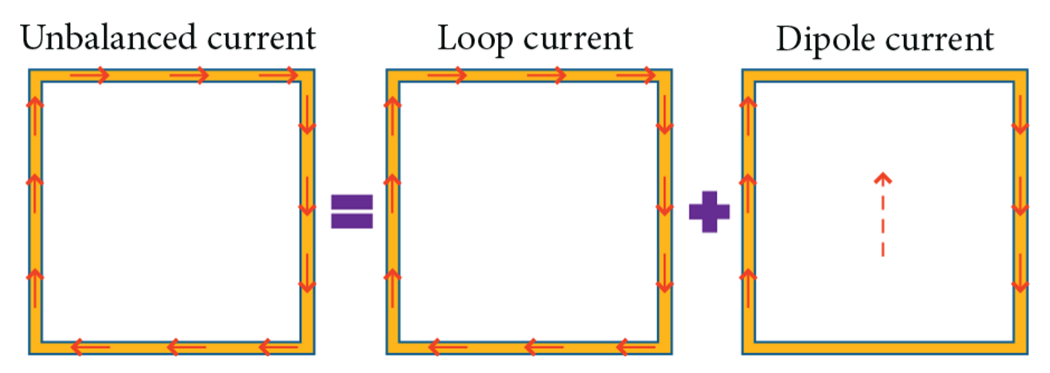
A nonuniform current distribution on a surface coil can be decomposed into the sum of a loop-like (divergence-free) and an electric-dipole-like (comprised of both divergence-free and curlfree current components) current pattern.

Here, we propose to deliberately create an asymmetric current distribution on a loop coil, by strategically using different capacitance values. It was shown that the resulting configuration can be represented as the sum of the uniform closed-path current pattern of a typical loop coil and an open-path current pattern that could be achieved with an electric dipole [25] (Figure 1). Our hypothesis is that this dual character could enable a hybrid loopole coil to achieve high performance at UHF, for cases in which the ideal current patterns predict that both loop-like and electric-dipole-like contributions are needed to approach the ultimate performance [14, 16]. The following sections detail the simulations and experiments performed to evaluate loopole in comparison to conventional loops and electric dipoles.

### 2.1. Simulations.

**Figure 2:**
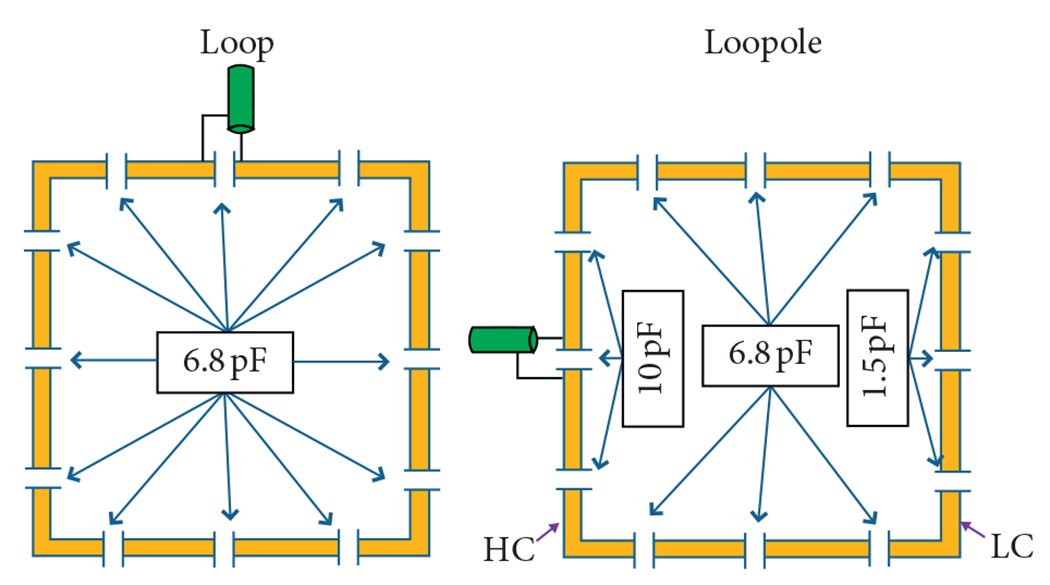
Electrical schematic of a typical (balanced) loop and a loopole element with the respective capacitance distribution.

Full wave electromagnetic simulations were performed with finite integration technique software (CST Microwave Studio, Providence, RI). A uniform cylindrical phantom was modeled with *ε_r_* = 81.8, *σ* = 0.60, 29.5 cm diameter, and 140 cm length to emulate a human body load. To demonstrate the proof-of-concept, one rectangular loopole (20 cm × 14.2 cm) was modeled in the simulation framework with 12 capacitors equally spaced around the perimeter. The capacitor values in the two vertical arms were asymmetrically chosen to create a high current arm (HC) and a low current arm (LC) on the loopole (Figure 2), while the capacitor values on two horizontal arms matched the capacitor values in the balanced loop. We then optimized the capacitance distribution for each arm by recording ${\text{B}}_{\text{1}}^{\text{+}}$ at the center of the phantom while the capacitor values in the high current arm of the loopole were increased and values in the low current arm were correspondingly lowered to maintain fixed resonant frequency. This process was repeated until the values in the low current arm required for resonance were approximately zero. For comparison, we also simulated a dipole antenna and a conventional loop in which the high and low current arms were balanced with a symmetric capacitor distribution. The loop had dimensions identical to those of the loopole, while the dipole was 36 cm long in the *z*-direction to achieve self-resonance. Each element was tuned, matched (<−25 dB), and excited with a 50 Ω port at 297.2 MHz, the operating frequency of 7 T UHF scanners. The excitation port was located at the service end for the loop and in the center of the high current arm for the loopole (Figure 2). The dipole was excited at the center.

To evaluate the performance of each element in an array configuration, eight loopoles conforming to a cylindrical surface of 31 cm in diameter were simulated. For comparison, an array of eight loops and an array of eight dipoles were also simulated. Individual coils in the loop and loopole arrays were overlapped with neighboring coils to minimize mutual inductance (<−14 dB coupling for all coil combinations, including those in the dipole array; <−20 dB matching for all elements). The dimensions, capacitance distribution, and location of the excitation ports of the loop, loopole, and dipole arrays were identical to those of the single elements described above. The coils were excited through 50 Ω ports with equal amplitude and with phases chosen for constructive interference at the center of the phantom.

The transmit efficiency was evaluated by computing ${\text{B}}_{\text{1}}^{\text{+}}$ maps normalized for 1 W of input power. The receive performance of the arrays was evaluated by optimally combining individual coil contributions and generating the corresponding SNR maps [26]. To demonstrate the loopole’s characteristic asymmetry and investigate its dependence on the port location, ${\text{B}}_{\text{1}}^{\text{+}}$ and SNR maps were generated with the main magnetic field oriented both in +*z* (orientation 1) and −*z* (orientation 2) directions. Peak 10 g SAR was calculated in the phantom volume to evaluate the SAR performance for each coil array.

### 2.2. Experiments.

To characterize the load sensitivity (resonant frequency shift), we measured reflection (S_11_) for a single loopole and a dipole as a function of distance to the phantom. To emulate the simulation setup, the loop, loopole, and dipole arrays were constructed on an acrylic tube with a 31.5 cm outer diameter. The loop and loopole coils were 20 cm × 14.2 cm and constructed of tinned bus wire (AWG12) incorporating twelve distributed capacitors. The dipoles were constructed from an FR4 circuit board with 7mm wide traces and lengths adjusted between 32 cm and 36 cm to fine-tune their resonant frequency. All coils were matched to 50 Ω (<−20 dB) with quarter-wavelength lattice baluns in the presence of a uniform cylindrical phantom (29.5 cm diameter and 120 cm length) filled with deionized water, 1.24 g/L NiSO_4_ × _6_H_20_, and 2.62 g/L NaCl, which provided a body-like load with *ε_r_* = 81.8 and *σ* = 0.6 s/m at 297.2 MHz (measured with a dielectric probe model 85070e, Agilent, Santa Clara, CA). The loop and loopole elements were optimally overlapped for geometric decoupling; the worst-case coupling between coils among all three topologies was −11 dB.

All imaging experiments were performed on a MAGNETOM 7 T scanner (Siemens Healthineers, Erlangen, Germany), equipped with an eight-channel parallel transmit system. Transmit phases were chosen to align at the center of the phantom. The excitation amplitude was calibrated at the center using a turbo flash scan with a preparation pulse [27]. ${\text{B}}_{\text{1}}^{\text{+}}$ maps were acquired for each of the constructed arrays using the AFI method [28]. Images in SNR units were calculated for each array using the method proposed by Kellman and McVeigh [26] from GRE acquisitions with and without RF excitation (TR/TE/FA/BW = 2000 ms/3.6 ms/90°/300 Hz per pixel, matrix = 64 × 64, FoV = 320 mm, slice thickness = 5 mm).

## 3. Results

### 3.1. Single-Element Simulations.

**Figure 3:**
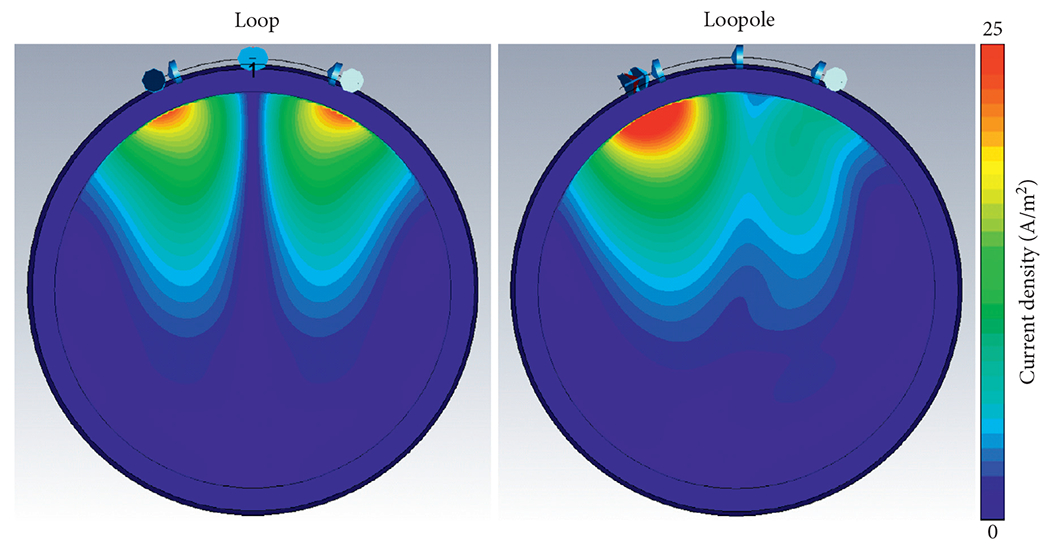
The current density produced by a loop and a loopole in the body-mimicking uniform phantom clearly indicates the balanced and unbalanced current characteristics of the two elements, respectively.

**Figure 4:**
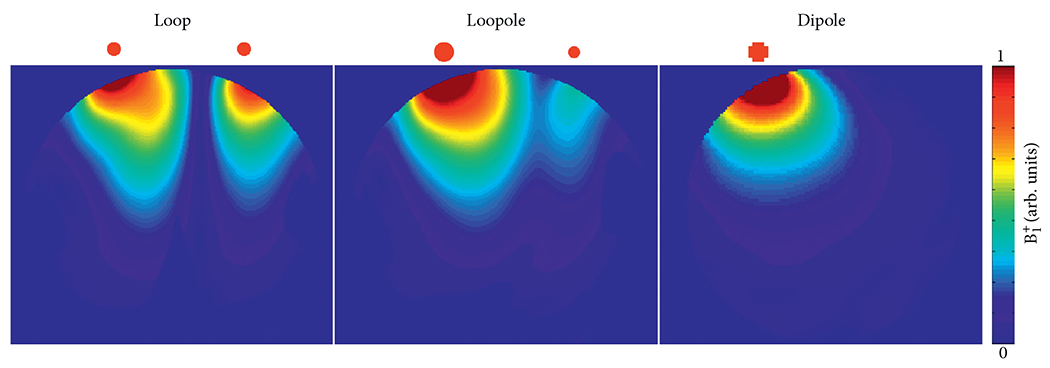
Single-element ${\text{B}}_{\text{1}}^{\text{+}}$ maps produced by loop, loopole, and electric dipole in simulations. The loop produces an asymmetric ${\text{B}}_{\text{1}}^{\text{+}}$ distribution with a strong null, whereas the electric dipole produces a nearly symmetric ${\text{B}}_{\text{1}}^{\text{+}}$. The loopole exhibits, instead, a hybrid behavior with the ${\text{B}}_{\text{1}}^{\text{+}}$ focused near the high current arm and with a weak null.

The optimized capacitance distribution for the simulated loopole resulted in a 3.4 to 1 ratio between the high and low current arms (Figure 2). This asymmetry in current distribution is illustrated in the current density maps in Figure 3, which compare the imbalanced current density below the opposing arms of the loopole and the balanced current density below the opposing arms of the traditional loop. Figure 4 shows the corresponding ${\text{B}}_{\text{1}}^{\text{+}}$ maps next to the ${\text{B}}_{\text{1}}^{\text{+}}$ of the electric dipole. The loop exhibits the characteristic ${\text{B}}_{\text{1}}^{\text{+}}$ asymmetry observed at UHF [6], with a strong null between the lobes, whereas the electric dipole exhibits a nearly symmetric ${\text{B}}_{\text{1}}^{\text{+}}$ distribution. The ${\text{B}}_{\text{1}}^{\text{+}}$ field produced by the loopole exhibits, instead, a hybrid behavior that seems to incorporate the behaviors of both the loop and the dipole. In fact, we can observe a lobe of strong magnetic field below the high current arm, which resembles the ${\text{B}}_{\text{1}}^{\text{+}}$ of the dipole, next to a low intensity band and a region of weak magnetic field below the low current arm, which resemble the null and the second lobe of the loop ${\text{B}}_{\text{1}}^{\text{+}}$.

### 3.2. Array Simulations.

**Figure 5:**
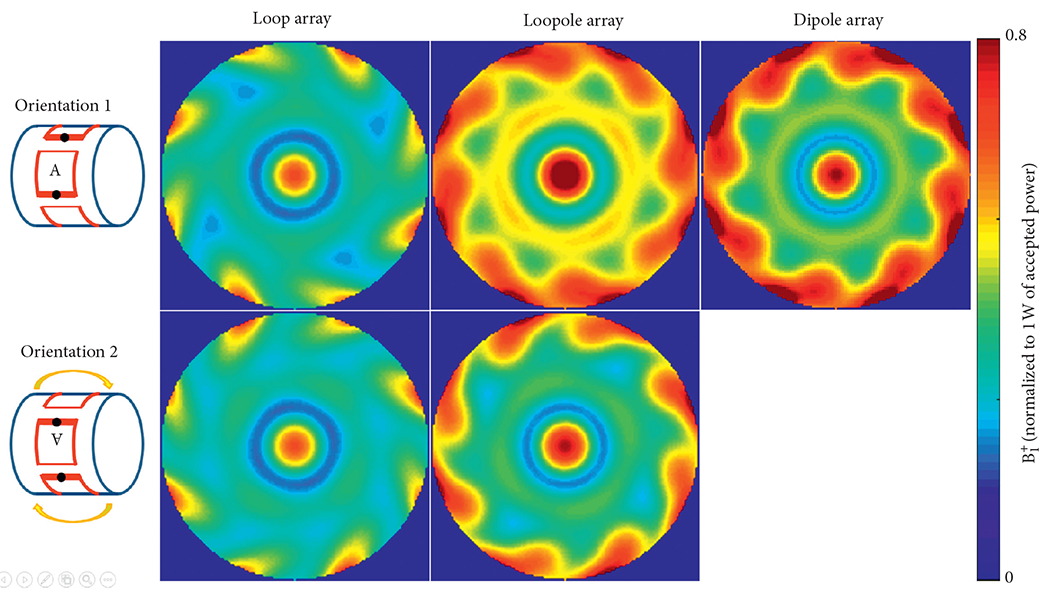
Simulated ${\text{B}}_{\text{1}}^{\text{+}}$ maps normalized to 1 watt of accepted power. A small deviation is observed between the ${\text{B}}_{\text{1}}^{\text{+}}$ maps produced by the loop array in the two orientations. The ${\text{B}}_{\text{1}}^{\text{+}}$ map for the dipole array is identical between the two orientations (hence, the orientation 2 map is not shown). The ${\text{B}}_{\text{1}}^{\text{+}}$ map for the loopole array is more markedly different between orientations. In the optimal orientation, the loopole array outperforms the loop array by 30% and the dipole array by 15% in central ${\text{B}}_{\text{1}}^{\text{+}}$.

**Figure 6:**
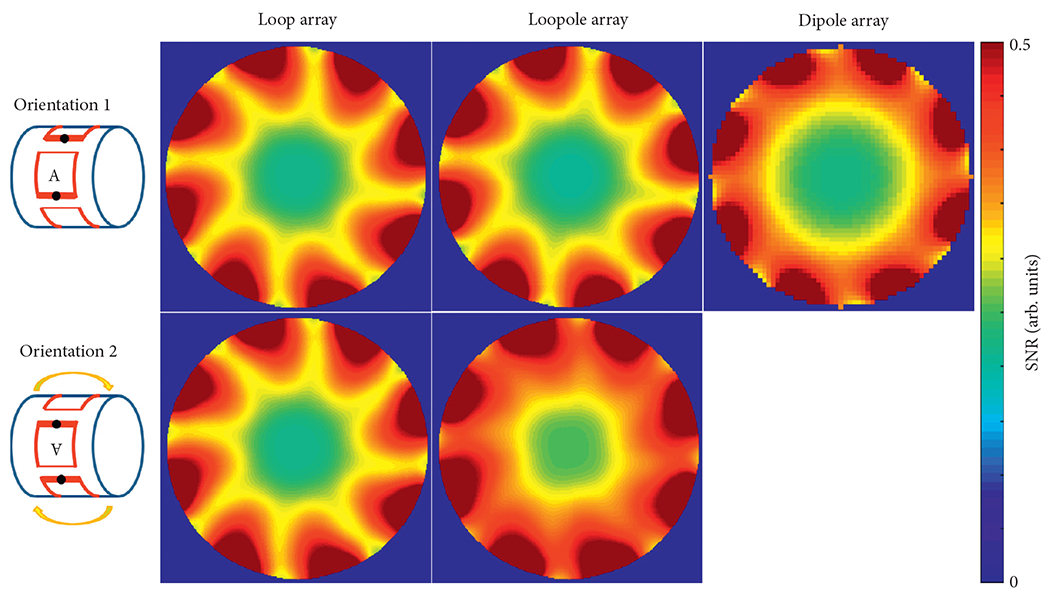
Simulated SNR maps show that, in the optimal orientation, the loopole array outperforms the loop array by 22% and the dipole array by 13% at the center.

**Figure 7:**
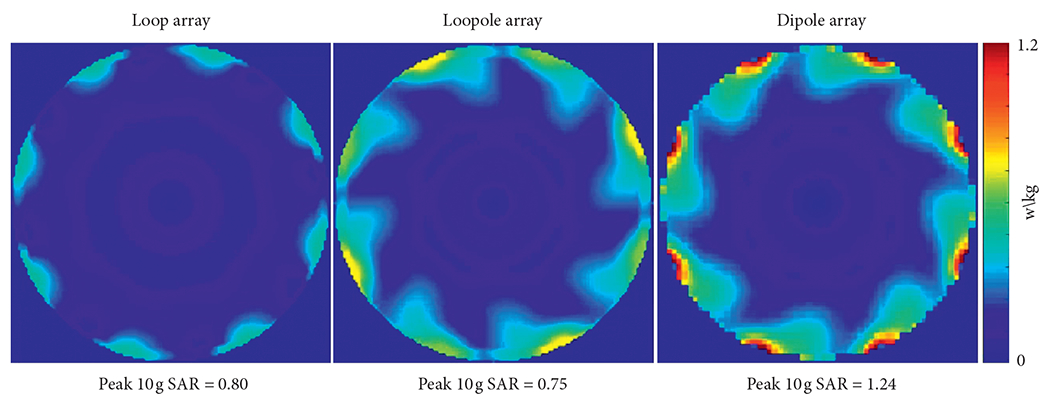
Simulated central axial peak 10 g SAR maps with peak SAR values indicate that the loopole array produced the lowest SAR among the designs compared, 40% lower than the dipole array. The SAR hot spots for the loopole array and the dipole array are located close to their corresponding excitation ports (center of the high current arm for the loopole and at the center for the dipole). The SAR hot spot for the loop array was located close to the excitation port (at the service end).

The performance of the different arrays was evaluated at the center of the phantom, where both loop-like and electric-dipole-like currents are needed to approach the ultimate performance at 7 T[15]. The transmit efficiency and SNR results at the central voxel obtained from the eight-channel array simulations are summarized in Table 1. Due to the symmetric nature of its current distribution, the loop array produced almost the same ${\text{B}}_{\text{1}}^{\text{+}}$ and SNR for both B_0_ orientations (Figures 5 and 6). The ${\text{B}}_{\text{1}}^{\text{+}}$ and SNR of the electric dipole array were plotted only for the case of B_0_ along the +*z* direction, because they are independent from the orientation. On the contrary, the loopole array exhibited an asymmetric behavior, producing a larger ${\text{B}}_{\text{1}}^{\text{+}}$ in orientation 1 compared to orientation 2. The opposite was observed in the receive case, where the loopole array achieved greater SNR in orientation 2 compared to orientation 1. When used in the optimal orientation, the loopole array outperformed the loop array by 31% and 22% and the dipole array by 15% and 13% in transmit efficiency and SNR, respectively. Central axial SAR maps are shown in Figure 7. In the optimal orientation, the loopole array produced the lowest 10 g peak SAR among the three configurations (40% lower compared to the dipole array).

### 3.3. Experiments.

**Figure 8:**
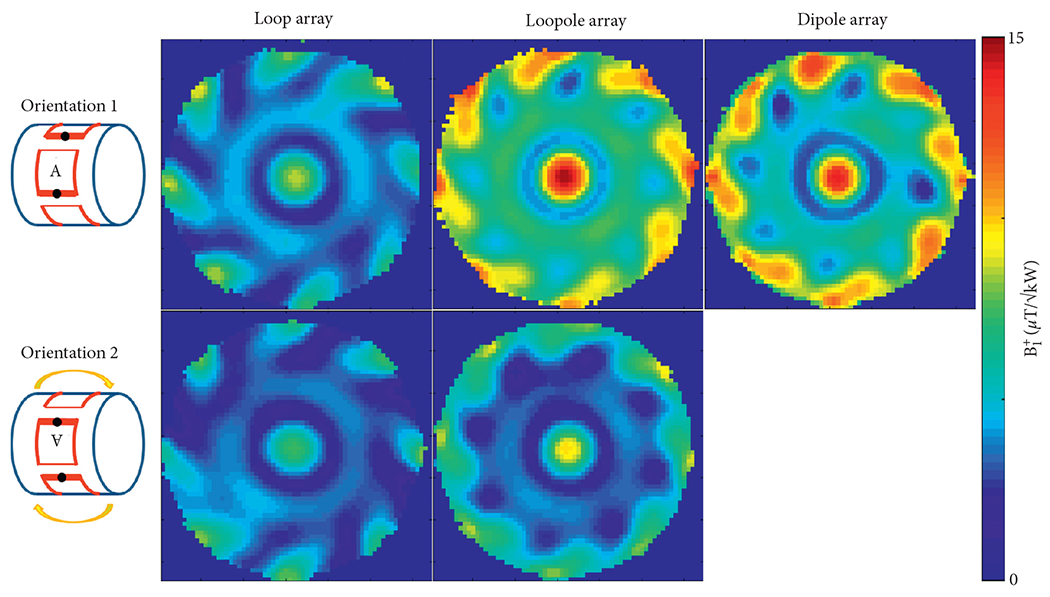
Experimental ${\text{B}}_{\text{1}}^{\text{+}}$ maps clearly show the asymmetric behavior of the loopole array. A small deviation in ${\text{B}}_{\text{1}}^{\text{+}}$ is observed in the loop array between the two orientations. In the optimal orientation, the loopole array demonstrated a 40% boost in central ${\text{B}}_{\text{1}}^{\text{+}}$ when compared to the loop array and a 5% boost when compared to the dipole array.

**Figure 9:**
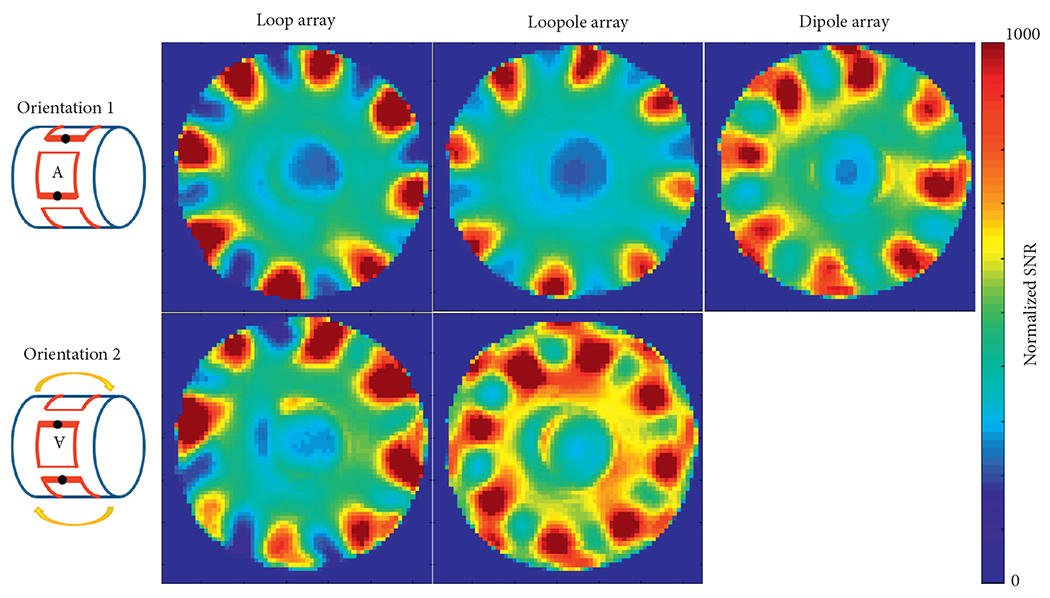
Experimental SNR maps again demonstrate the asymmetric behavior of the loopole array. In the optimal orientation, the loopole array demonstrated a 25% boost in the central SNR when compared to the loop array and 9% boost when compared to the dipole array.

The unloaded and loaded *Q* values were 80 and 6 for the loop and 55 and 6 for the loopole, with the corresponding *Q* ratios of 13 and 9, indicating sample noise dominance. The experimental ${\text{B}}_{\text{1}}^{\text{+}}$ and normalized SNR maps (Figures 8 and 9 show good qualitative agreement with the simulations.

**Figure 10:**
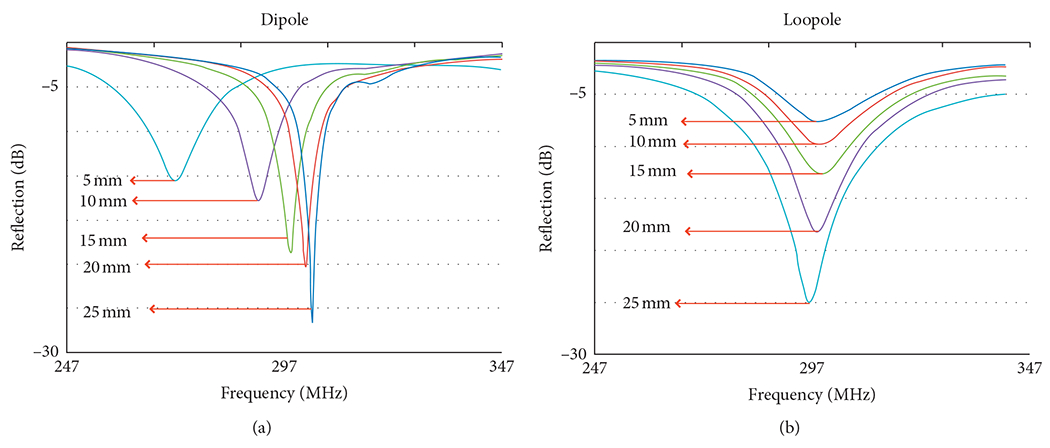
Reflection (S_11_) measurements demonstrate a 30 MHz shift in the resonant frequency of a dipole when its distance to the phantom varied from 25 mm to 5 mm. The frequency response of the loopole was much more stable in this regard with a 3 MHz frequency shift.

Reflection (S_11_) measurements (Figure 10) show that the dipole exhibited a 30 MHz resonant frequency shift when its distance to the sample changed from 25 to 5 mm. In comparison, the loopole shifted by 3 MHz.

## 4. Discussion

We have revisited the loopole coil, which is a resonant loop deliberately designed to have an asymmetric current distribution to capture both loop-like and electric-dipole-like currents. The loopole array demonstrated improved SNR or ${\text{B}}_{\text{1}}^{\text{+}}$ compared to arrays composed of either loops or electric dipoles alone at 7 T. The outcome was predictable with respect to the balanced loop, which can only capture the divergence-free contribution to the ultimate intrinsic SNR.

While the loopole slightly outperformed the electric dipole array, which also is comprised of both divergence-free and curl-free currents, it should be noted that we utilized basic self-resonant dipoles for the comparison. In fact, much progress has been made recently to optimize electric dipole performance in terms of SAR and SNR [29–36]. At the same time, we empirically selected a 3.4 :1 current ratio between the conductive arms of the loopole to demonstrate the proof-of-concept, but the design space was by no means exhausted and further optimization could be possible. For example, one can imagine utilizing the extra degrees of freedom in capacitor distribution to tailor transmit or receive sensitivity for a specific depth, based on the anatomy of interest [37].

Yan et al. recently showed that nonuniform loop current distributions could be exploited for coil decoupling by altering the balance between the horizontal arm (opposite to the drive port) and the rest of the loop [24]. In one example, they reduced the horizontal arm capacitance by a factor of ~20 with respect to a balanced loop to create a self-decoupled coil. In comparison, we varied the current balance between the vertical arms to optimize ${\text{B}}_{\text{1}}^{\text{+}}$ and utilized geometric overlap to decouple neighbor coils. While ${\text{B}}_{\text{1}}^{\text{+}}$ may represent a more pertinent optimization metric compared to decoupling, we want to point out that unbalanced vertical arms in the loopole imply a preferential main magnetic field direction. Thus, a loopole array can be arranged to outperform arrays of traditional loops or electric dipoles in either excitation or reception. Our results suggest that the loopole should not be used as a transmit/receive element. In fact, due to its asymmetric nature, it is not possible to simultaneously optimize the loopole capacitor distribution for transmit and receive applications.

We found that the distributed capacitance in a loopole makes its resonant frequency less load-dependent than for an electric dipole. This property makes the loopole easier to tune and match and could reduce transmit power requirements over a range of loading conditions. On the other hand, an asymmetric capacitor distribution necessarily results in asymmetric electrical field distribution and heating [38]. Therefore, it is essential to carry out electromagnetic field simulations to determine local SAR for a specific capacitor distribution to ensure compliance with International Electrotechnical Commission guidelines.

Given the recent popularity of mixed loop/dipole arrays [21, 22], we were interested in comparing their performance to a loopole array. Our simulated results showed that an eight-channel loopole array produced nearly identical transmit efficiency and 38% lower peak SAR than a 16-channel mixed array with eight loop/dipole pairs (Figures S1 and S2), despite one-half the number of transmit channels. On the receive side, the mixed array significantly outperformed the loopoles in the periphery, while the loopoles provided 89% of the SNR as the mixed array at the center (Figure S3). While not investigated here, it is likely that the mixed array, by leveraging its increased channel count owing to orthogonal fields sensitized by the loop/dipole pairs, would also outperform the loopole in terms of parallel imaging performance. While this preliminary evidence suggests that loopole arrays may fall short of replacing mixed arrays, they may find application as a transmit-only device on systems with limited channels.

In conclusion, we investigated the performance of a recently introduced hybrid coil element that supports both loop-like and electric-dipole-like currents, by employing a counter-intuitive strategy of a highly unbalanced current distribution around a closed conductor path.

## Supplementary Material

Supplementary Materials

## Figures and Tables

**Table 1:** Simulated and experimental ${\text{B}}_{\text{1}}^{\text{+}}$ and SNR values at the central voxel in the body-sized phantom. Both in simulations and experiments, the loopole array demonstrates significant ${\text{B}}_{\text{1}}^{\text{+}}$ efficiency and SNR boosts compared to the loop array and marginal improvements compared to the dipole array.

		Loop		Loopole		Dipole		Figure
Orientation		1	2	1	2	1	2	
Simulation	${\text{B}}_{\text{1}}^{\text{+}}$	0.65	0.64	0.94	0.75	0.79	N/A	5
	SNR	0.21	0.21	0.21	0.25	0.22	N/A	6

Experiment	${\text{B}}_{\text{1}}^{\text{+}}$(uT/VkW)	8.56	7.43	14.74	9.70	13.98	N/A	8
	SNR	4.76	5.21	4.20	6.82	6.2	N/A	9
