# Supplementary material for: The “Loopole” Antenna: A Hybrid Coil Combining Loop and Electric Dipole Properties for Ultra-High-Field MRI
Source: Concepts Magn Reson Part B Magn Reson Eng. Author manuscript; Available in PMC 2021 Jun 16. (PMC8207246; doi:10.1155/2020/8886543)
Supplement: Supplementary Materials [file NIHMS1708967-supplement-Supplementary_Materials.docx]

**Methods**

We compared two eight-channel loopole arrays to a 16-channel mixed array (eight loops and eight dipoles using full wave electromagnetic simulations with finite integration technique software (CST Microwave Studio, Providence, RI). Preliminary simulations were performed on a balanced loop and single dipole to determine the coil lengths that maximized B_1_^+^ for 1 W input power at the center of the phantom described in the manuscript. The balanced loop was subsequently transformed into a loopole by adjusting the capacitor distribution to further maximize B_1_^+^.

Guided by the preliminary results, we then simulated two eight-channel loopole arrays whose elements were 16.75 cm in length. The two arrays were identical except for the loopole orientation with respect to B_0_; one was orientated to maximize transmit efficiency and the other for receive efficiency (see Figures 5 and 6). The loopoles had an arc length of 14.2 cm in order to overlap and decouple nearest neighbors. The loopole arrays were compared to a 16-channel mixed array in which dipoles (18.75 cm) bisected balanced loops (14.2 x 16.75 cm) to form eight loop/dipole pairs.

The coils were excited through 50 Ω ports with equal amplitude and with phases chosen for constructive interference at the center of the phantom. The transmit efficiency, SNR, and SAR were evaluated using the methods described in the manuscript.

**Results**

With the loopole array in transmit orientation, it exhibited nearly identical transmit efficiency (within 1%) as well as 38% lower peak 10g SAR compared to the16 channel mixed array (Fig. S1 and S2), despite having half the number of elements. With the loopole array in receive orientation, it provided 89% of the SNR at the center of the phantom as the mixed array (Fig. S3). As expected, the mixed array provided higher SNR gains at shallow depths (21% at 7.5 cm) when compared to the loopole array due to higher channel count.


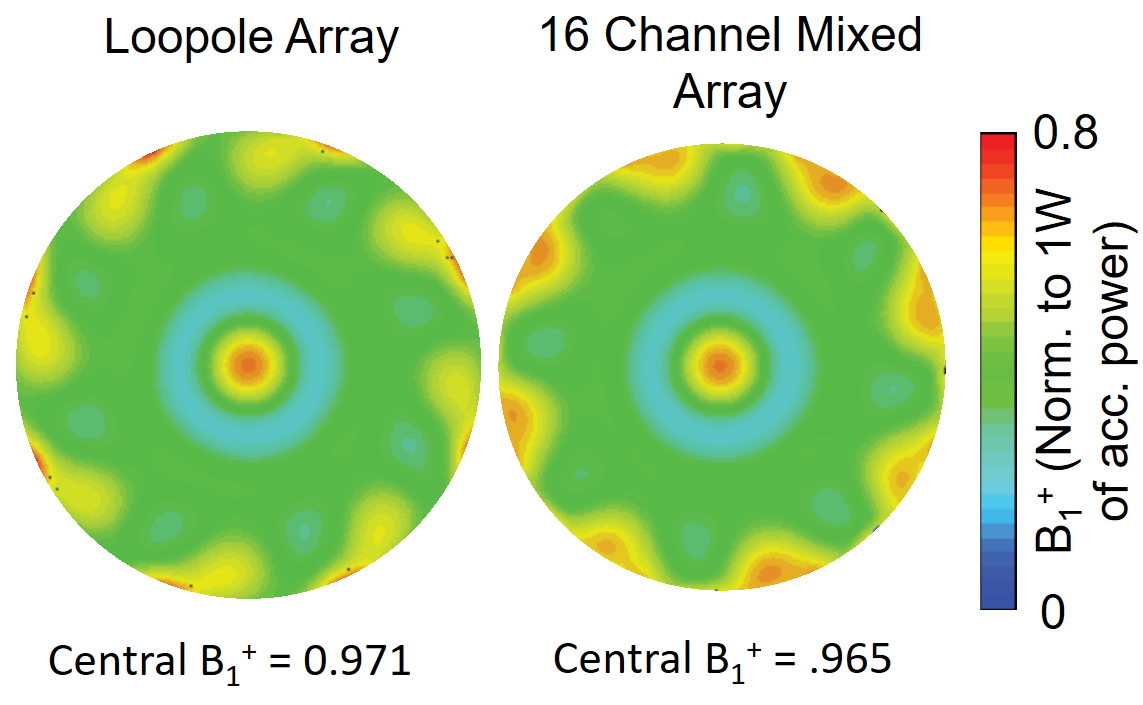


**Figure S1**

Simulated B_1_^+^ maps normalized to 1 Watt of accepted power. The loopole array in transmit orientation exhibits nearly identical transmit efficiency as the mixed loop-dipole array.


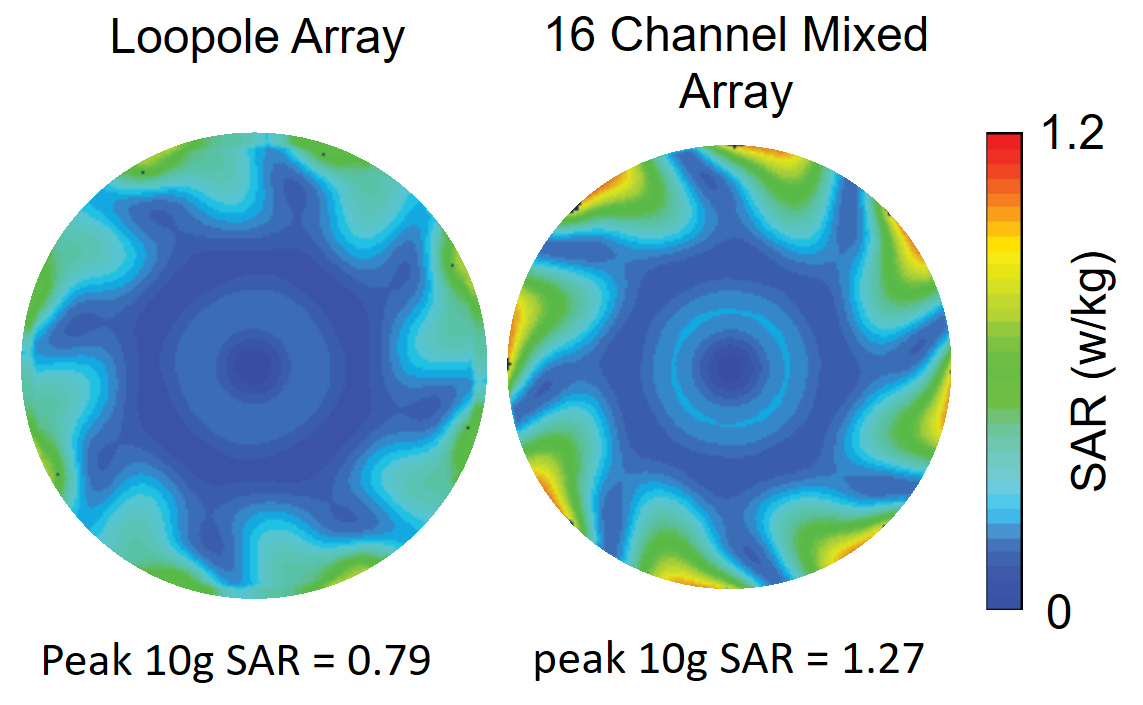


**Figure S2**

Simulated central axial peak 10g SAR maps with peak SAR values indicate that the loopole array produced 38% lower SAR than the mixed loop/dipole array.

**
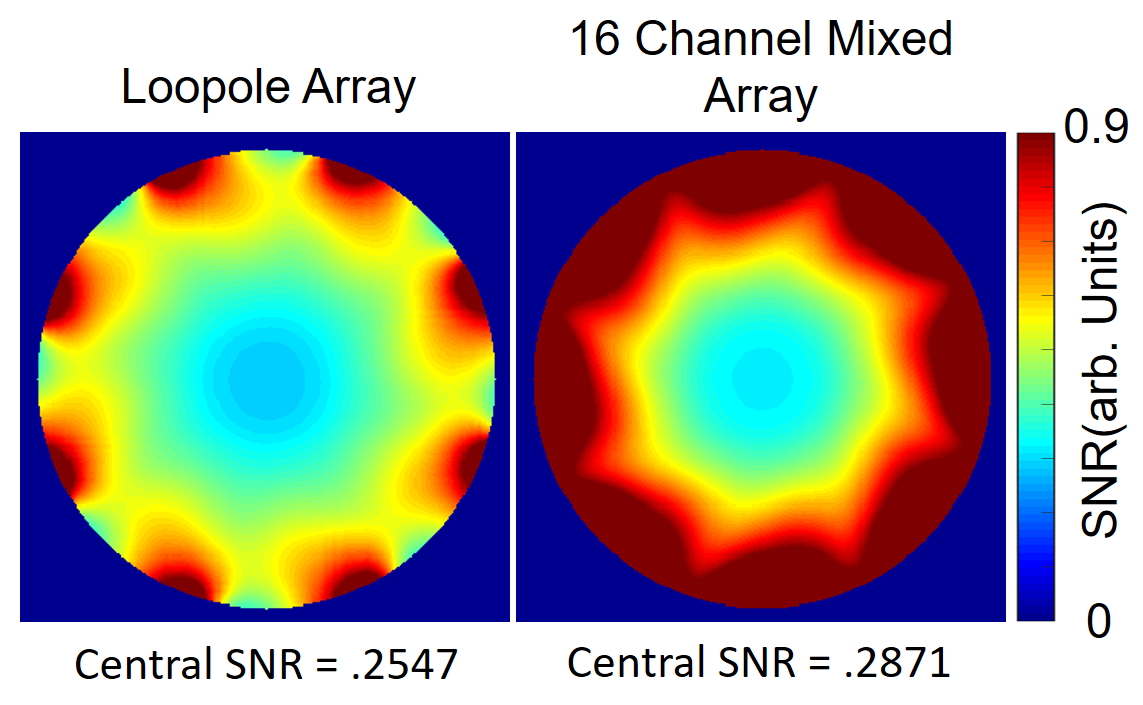
**

**Figure S3**

Simulated SNR maps show that the 16-channel mixed loop/dipole array outperforms the eight-channel loopole array in receive orientation by 11% at the center.
